# MiR-146b Mediates Endotoxin Tolerance in Human Phagocytes

**DOI:** 10.1155/2015/145305

**Published:** 2015-09-14

**Authors:** Tiziana Ada Renzi, Marcello Rubino, Laura Gornati, Cecilia Garlanda, Massimo Locati, Graziella Curtale

**Affiliations:** ^1^Department of Medical Biotechnologies and Translational Medicine, University of Milan, Milan, Italy; ^2^Humanitas Clinical and Research Center, Via Manzoni 113, 20089 Rozzano, Italy; ^3^Department of Cancer Biology, The Scripps Research Institute, Jupiter, FL 33458, USA

## Abstract

A proper regulation of the innate immune response is fundamental to keep the immune system in check and avoid a chronic status of inflammation. As they act as negative modulators of TLR signaling pathways, miRNAs have been recently involved in the control of the inflammatory response. However, their role in the context of endotoxin tolerance is just beginning to be explored. We here show that miR-146b is upregulated in human monocytes tolerized by LPS, IL-10, or TGF*β* priming and demonstrate that its transcription is driven by STAT3 and RUNX3, key factors downstream of IL-10 and TGF*β* signaling. Our study also found that IFN*γ*, known to revert LPS tolerant state, inhibits miR-146b expression. Finally, we provide evidence that miR-146b levels have a profound effect on the tolerant state, thus candidating miR-146b as a molecular mediator of endotoxin tolerance.

## 1. Introduction

Deregulation of the inflammatory response contributes to tissue damage in several pathological conditions, including autoimmune and infectious diseases [1–3]. To balance inflammation, the innate immune system has developed a regulatory mechanism by which innate immune cells, monocytes, and macrophages in particular display reduced response to subsequent challenges after they have been exposed to low concentrations of endotoxin (e.g., LPS) [4, 5], entering in a so-called “tolerant” state. Although endotoxin tolerance has been considered as a protective mechanism to regulate overexuberant inflammation, the incidence of endotoxin tolerance has been associated with high risk of secondary infections and an endotoxin-tolerant state is considered to be a clinical phenomenon observed not only in sepsis [6, 7] but also in diseases like acute coronary syndrome [8] and cystic fibrosis [9, 10], and in these pathologies the risk of new infections correlates with a refractory state of innate immune cells.

Much of our knowledge on the molecular mechanisms responsible for endotoxin tolerance arises from* in vitro* studies, where monocytes primed with low doses of LPS or with IL-10 or TGF*β* become tolerant to a subsequent LPS challenging and strongly reduce their production of TNF*α* and other proinflammatory cytokines [11]. IFN*γ* represents a key negative regulator of LPS tolerance, being able to revert tolerance and restore TNF*α* production both in* in vitro* and* in vivo* models [12]. The molecular basis of endotoxin tolerance has not been completely elucidated, but it is now clear that it is a dynamic process implying a profound gene reprogramming [13, 14]. In particular extensive studies demonstrated the impairment of the Toll-like receptor (TLR) signaling pathway at multiple levels, with the consequent repression of proinflammatory mediators (i.e., TNF*α*, IL-6, and IL-12), and the concomitant upregulation of anti-inflammatory molecules, such as IL-10 and TGF*β* [15]. Functionally, tolerant monocytes also exhibit increased phagocytosis due to increased expression of CD64 and impaired antigen presentation ability due to the downregulation of major histocompatibility class II (MHC II), CD86, and class II transactivator (CIITA) [9, 16, 17].

A growing number of miRNAs have been reported to be involved in the regulation of the inflammatory response [18–27], but only recently studies described the differential expression and effects of miRNA in the context of endotoxin tolerance [28, 29]. MiR-146a was the first miRNA described as upregulated in tolerant THP-1 monocytic cells after priming with low dose of LPS and was shown to partially induce LPS desensitization in monocytes [28, 29]. Evidence suggesting a possible role of miR-155 and miR-125b in tolerance has also been reported [30]. It is still unclear to which degree each miRNA contributes to the development of endotoxin tolerance, but of note all these miRNAs have been shown to modulate TLR4 signaling pathway by targeting different components of its signaling cascade [31]. In the present study we show the involvement of miR-146b in the induction of endotoxin tolerance by showing its upregulation in LPS tolerant monocytes, its induction by the anti-inflammatory stimuli IL-10 and TGF*β*, and its negative regulation by IFN*γ*. We also demonstrate that tuning miR-146b expression results in the induction or reversion of endotoxin tolerance in the THP-1 monocytic cell line.

## 2. Materials and Methods

### 2.1. Reagents

LPS was from* E. coli* (serotype 055:B5). IL-10, IL-4, IL-13, and TGF*β* were from R&D system; IFN*γ* was from Peprotech; dexamethasone was from Sigma Aldrich. Antibodies anti-Pol II (N-20), anti-RUNX3 (H-50), and anti-STAT3 (C-20) for ChIP experiments were purchased from Santa Cruz Biotechnology and anti-Ago2 was purchased from Abcam.

### 2.2. Cell Purification and Culture

Human monocytes were obtained from healthy donor buffy coats by two-step gradient centrifugation using Ficoll (Biochrom) and Percoll (Amersham) followed by incubation of purified cells in RPMI 1640 (Lonza) without serum for 10 min at 37°C with 5% CO_2_. Adherent monocytes were washed twice with PBS and then cultured with RPMI medium supplemented with 10% FBS and L-glutamine as fully described below. The purity of the monocytes cultures was tested by CD14 staining and flow cytometry analysis, with an average of 90% CD14^+^ cells.

Monocytes and THP-1 cells (ATCC) were grown in RPMI supplemented with 10% heat-inactivated fetal bovine serum (FBS; Lonza), 100 U/mL penicillin/streptomycin (Lonza), and 25 mM L-glutamine (Lonza) at 37°C with 5% CO_2_. HEK-293T cells (ATCC) were grown in D-MEM (Cambrex) supplemented with 10% FBS, 100 U/mL penicillin/streptomycin, and 25 mM L-glutamine at 37°C with 5% CO_2_.

### 2.3. Chromatin Immunoprecipitation (ChIP) Assay

ChIP experiments were performed as described elsewhere [32]. 10^7^ human purified monocytes were culture and extracted DNA was used to perform qPCR using promoter-specific primers [21]. 1% of starting chromatin was used as input. Signals obtained from the ChIP samples were normalized on those obtained from the corresponding input samples, according to the formula: 100 × 2^∧(input  Ct − sample  Ct)^. Results were expressed as fold enrichment relative to untreated cells.

### 2.4. Quantification of miR-146b Expression

Total RNA was purified using TRIzol Reagent (Ambion) and extracted with “Directzol RNA miniprep” kit (Zymo Research). Q-PCR was conducted using a 7900HT Real-time PCR System. 100 ng of total RNA was reverse transcribed for quantification of miR expression using TaqMan MiRNA Reverse Transcription kit (Applied Biosystems), according to manufacturer's instructions and as previously described [20, 21]. MiRNA expression values were calculated according to the comparative threshold cycle method, using the ubiquitous small nucleolar RNA U6 as endogenous reference.

### 2.5. Construct Generation

To overexpress miR-146b in THP-1 monocytic cells a lentiviral-based system was used, as described elsewhere [21] Briefly, the miR/lentiviral-based expression vector pRRL-miR-146b was generated by cloning in the pRRLSIN.cPPT.PGK-GFP.WPRE vector (plasmid #12252; Addgene) a 500 bp region encompassing the pre-miR-146b. The lentiviral construct pRRL-ct, encoding for a hairpin yielding a 22-mer RNA with no homology to any human gene, was used as mock construct. THP-1 cells were transduced with pRRL-146b or pRRL-ct vectors and pRRL-146b THP-1 GFP^+^ cells and pRRL-ct THP1 GFP^+^ cells were sorted by FACS with a 90–95% of purity. To knockdown miR-146b-5p expression, THP-1 cells were transduced with the miRzip lentivector-based construct anti-miR-146b and the relative control (System Biosciences) [21]. Transduced GFP^+^miRT-146b and GFP^+^miRT-ct THP-1 cells were sorted by FACS with over a 95% of purity.

### 2.6. Immunoprecipitation of Ago2-Bound RNAs

Immunoprecipitation of Ago2-bound RNAs (RIP) was performed as previously described [21]. 30 × 10^6^ stimulated monocytes were used and results were expressed as fold enrichment relative to unstimulated samples.

### 2.7. ELISA Assay

All antibodies and detection reagents were purchased from R&D Systems. The ELISA was carried out according to the manufacturer's instructions. Samples were diluted so that the optical density fell within the optimal portion of a log standard curve.

### 2.8. LPS Desensitization and Reversal

MiRT-ct and miRT-146b-5p THP1 cells were cultured in 24-well plates in 500 *μ*L RPMI supplemented with 10% FBS and 1% L-glutamine, incubated or not for 18 h with 50 ng/mL TGF*β* or 20 ng/mL IL-10, followed by LPS stimulation with 0.1 ng/mL or 20 ng/mL LPS. Cells were then washed twice and challenged with 10 ng/mL LPS. Supernatants were collected 24 h after stimulation.

### 2.9. Statistical Analysis

Statistical evaluation was determined using either Student *t*-test or one-way ANOVA and *p* values are reported in figures (^*∗*^ *p* < 0.05; ^*∗∗*^ *p* < 0.01).

## 3. Results

### 3.1. MiR-146b Expression Is Induced by IL-10 and TGF*β* in Human Primary Monocytes

**Figure 1:**
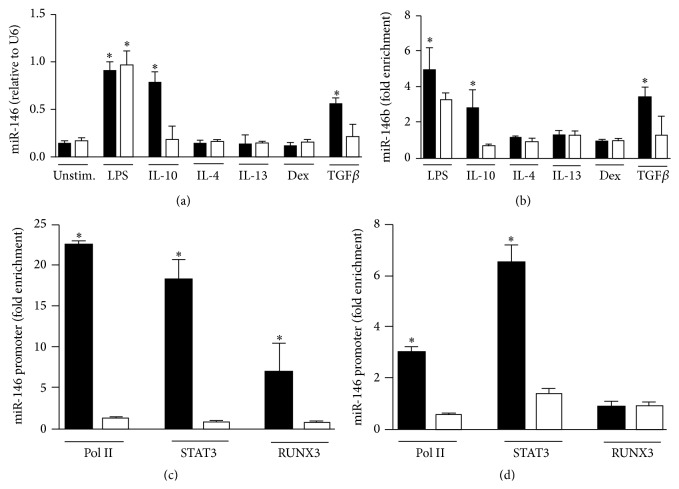
*MiR-146b is induced in human monocytes by TGFβ signaling pathway*. Human purified monocytes were stimulated for 24 h with 100 ng/mL LPS, 20 ng/mL IL-10, 20 ng/mL IL-4, 20 ng/mL IL-13, 20 ng/mL Dex, or 50 ng/mL TGF*β* and (a) miR-146b (black columns) and miR-146a (white columns) levels from total RNA were measured by qPCR in triplicate samples. (b) Cell extracts were subjected to RIP assay using anti-Ago2 or IgG control Abs and levels of miR-146b (black columns) and miR-146a (white columns) were measured by qPCR in triplicate samples. Results are expressed as fold change over control (mean ± SEM, *n* = 3). (c-d) ChIP assays were carried out on human purified monocytes stimulated or not for 4 h with 50 ng/mL TGF*β* (c) or 20 ng/mL IL-10 (d) using anti-Pol II, anti-STAT3, or anti-RUNX3 antibodies. Q-PCR was carried out using specific primers for miR-146b (black columns) or miR-146a (white columns) promoters. Results are expressed as fold change over control (mean ± SEM, *n* = 3).

The miR-146 family is composed by miR-146a and miR-146b, both of which are induced during the inflammatory response and target different component of TLR signaling pathway, thus acting as anti-inflammatory regulators [18, 21, 33]. In previous studies we reported that LPS induced the expression of both miR-146a and miR-146b in human monocytes [20] and demonstrated that miR-146b but not miR-146a induction was dependent on the endogenous production of IL-10 subsequent to LPS exposure [21]. We here investigated the regulation of miR-146b and miR-146a by anti-inflammatory mediators known to negatively modulate the activation of monocyte/macrophages. We found that, in addition to IL-10, TGF*β* was able to specifically increase the expression of miR-146b but not miR-146a. Glucocorticoids (Dex), IL-4, and IL-13 did not affect miR-146b and miR-146a expression levels (Figure 1(a)).

To investigate the functional activity of miR-146b we evaluated its presence into the Ago2-RISC complex and consistent with the expression data we found an enrichment of miR-146b only when monocytes were treated with LPS, IL-10, or TGF*β* (Figure 1(b)). As a control, we measured the relative enrichment of miR-146a and consistent with previous data [21] we detected miR-146a in Ago2-RISC complex only when monocytes were stimulated with LPS (Figure 1(b)).

To elucidate the transcriptional regulation of miR-146b, the* cis*-regulatory elements in the miR-146b promoter region were characterized. Monocytes exposed to TGF*β* or IL-10 showed an enrichment of Pol II onto the miR-146b promoter but not on miR-146a promoter (Figures 1(c) and 1(d)). Bioinformatic analysis performed on the 1 kbp promoter region of miR-146b showed the presence of two consensus sites for STAT3 and one for RUNX3. Since we previously demonstrated the binding of STAT3 to miR-146b promoter in IL-10 stimulated monocytes [21] (Figure 1(d)) we thought to determine whether STAT3 is also recruited to the miR-146b promoter region upon monocytes TGF*β* stimulation. We found that recruitment of STAT3 is specific to miR-146b but not to miR-146a promoter (Figure 1(c)). Interestingly, STAT3 has been reported to cooperate with RUNX3 [34], which is also directly induced by TGF*β* signaling pathway [35]. Given the proximity of the STAT3 and RUNX3 consensus sites on the miR-146b promoter region, we performed ChIP experiments and found the specific recruitment of RUNX3 transcription factor to the miR-146b promoter region only in monocytes challenged with TGF*β* (Figures 1(c) and 1(d)). Taken together, these data identify miR-146b as an IL-10- and TGF*β*-responsive gene, whose expression in monocytes is specifically driven by STAT3 in response to IL-10 and by STAT3 and RUNX3 in response to TGF*β*.

### 3.2. miR-146b Is Induced in Endotoxin Tolerized Human Monocytes

**Figure 2:**
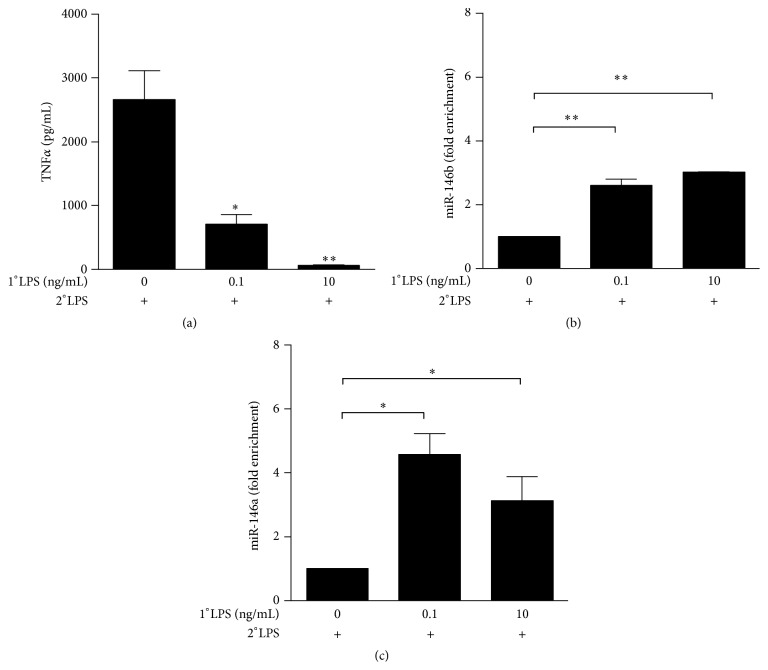
*miR-146b and miR-146a are induced in endotoxin tolerized monocytes*. Monocytes were first primed with 0, 0.1, or 10 ng/mL LPS (1°LPS) for 18 h and then challenged for 24 h with 10 ng/mL LPS (2°LPS). After 24 h stimulation, TNF*α* levels (a) were measured by ELISA in cell-free supernatants, and levels of (b) miR-146b and (c) miR-146a were assayed by qPCR in triplicate samples, normalized relative to U6, and expressed as fold enrichment as compared to unstimulated samples. Results are expressed as mean ± SEM of three independent experiments.

Since miR-146a was shown to be involved in LPS tolerance [28, 29] and we previously demonstrated that miR-146b is a negative regulator of TLR signaling [21], we investigated the role of miR-146b in the induction of LPS tolerance. We found TNF*α* strongly downregulated in* in vitro* tolerized monocytes (Figure 2(a)), in agreement with previous reports [36]. Interestingly, the decrease of TNF*α* levels was proportional to the dose of LPS used to prime cells: its expression was dramatically impaired in monocytes primed with 0.1 ng/mL LPS and completely abolished with 10 ng/mL LPS (Figure 2(a)). Next we analyzed miR-146b expression levels in tolerized monocytes, using miR-146a as positive control [28]. Strikingly, both miR-146b and miR-146a showed significant higher expression in tolerized monocytes compared to untolerized control cells (Figures 2(b) and 2(c), resp.), thus suggesting miR-146b putative role in LPS tolerance.

### 3.3. IL-10 and TGF*β* Priming Induced Tolerance in Human Monocytes and LPS Responsiveness Can Be Restored by IFN*γ* Pretreatment

**Figure 3:**
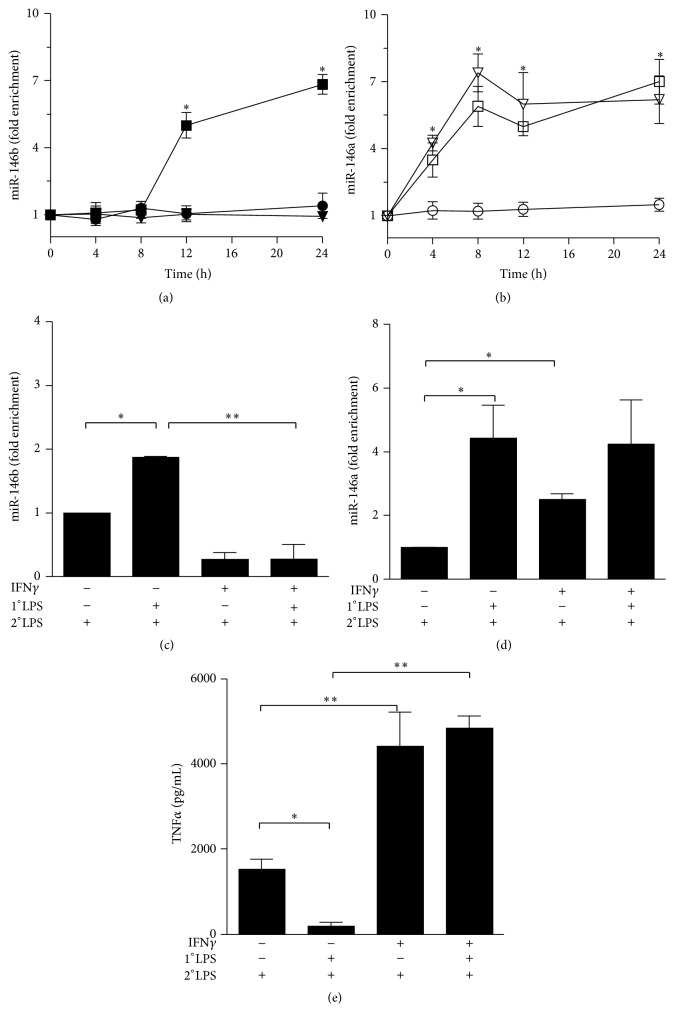
*IFNγ inhibits the upregulation of miR-146b in tolerized monocytes*. (a and b) Monocytes were cultured for the indicated times with 100 ng/mL LPS (circle), 20 ng/mL IFN*γ* (square), or both stimuli (triangle). (a) MiR-146b and (b) miR-146a were quantified by qPCR. (c to e) Human purified monocytes pretreated or not with 10 ng/mL IFN*γ*, primed with 0.1 ng/mL LPS (1°LPS) for 18 h, and then challenged with 10 ng/mL LPS (2°LPS). After 24 h stimulation levels of (c) miR-146b and (d) miR-146a were assayed by qPCR in triplicate samples, normalized relative to U6, and expressed as fold enrichment as compared to unstimulated samples. (e) TNF*α* levels were measured by ELISA in cell-free supernatants. Results are expressed as mean ± SEM of three independent experiments.

IL-10 and TGF*β* act as feedback inhibitors of the LPS-mediated inflammatory response and are part of a more complex network of regulation in which IFN*γ* operates an opposite function working as a coactivator of the inflammatory pathway triggered by LPS [37]. It has also been reported that IFN*γ* is able to revert LPS tolerance both in human [11, 38] and murine [39, 40] phagocytes. Accordingly, in human monocytes IFN*γ* pretreatment strongly enhanced LPS-dependent induction of TNF*α* and significantly impaired LPS-dependent tolerization effect (Figure 3(e)). Interestingly, IFN*γ* was able to inhibit the LPS-dependent induction of miR-146b when monocytes were challenged with both LPS and IFN*γ* (Figure 3(a)), whereas it did not affect the expression of miR-146a (Figure 3(b)). Moreover, pretreatment with IFN*γ* completely abolished the increased expression of miR-146b observed in LPS-tolerized monocytes, but it had no effect on the overexpression of miR-146a observed in tolerized monocytes (Figures 3(c) and 3(d), resp.).

**Figure 4:**
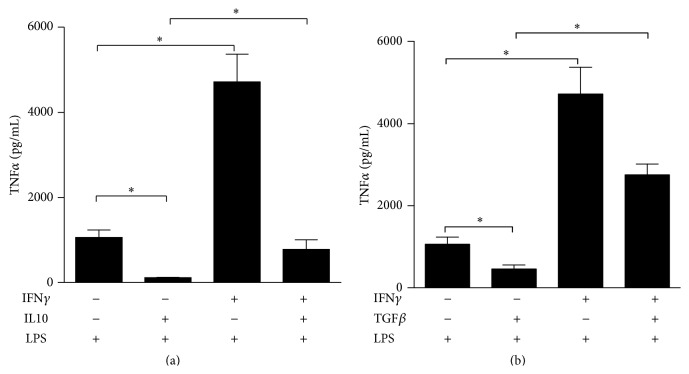
*Inhibitory role of IFNγ on IL-10- and TGFβ-dependent endotoxin tolerance*. Monocytes were pretreated or not with 10 ng/mL IFN*γ* and primed with 10 ng/mL IL-10 (a) or 50 ng/mL TGF*β* (b) for 18 h and then stimulated with 10 ng/mL LPS. Supernatants were collected 24 h later; TNF*α* protein levels were quantified by ELISA. Results are expressed as mean ± SEM of three independent experiments.

Opposite to IFN*γ*, the anti-inflammatory cytokines IL-10 and TGF*β* support LPS desensitization and have been experimentally used to induce hyporesponsiveness to LPS [11]. Both stimuli decreased the expression of TNF*α* induced by a following stimulation with LPS. IFN*γ* pretreatment, which strongly enhanced LPS-dependent induction of inflammatory cytokines, was also able to impair the IL-10- and TGF*β*-dependent tolerization effect (Figures 4(a) and 4(b)). Since we have observed that both IL-10 and TGF*β* induce the expression of miR-146b (Figure 1(c)), altogether these data candidate miR-146b as an effector of LPS tolerance.

### 3.4. Upregulation of miR-146b Mimics LPS Priming to Induce Endotoxin Tolerance

**Figure 5:**
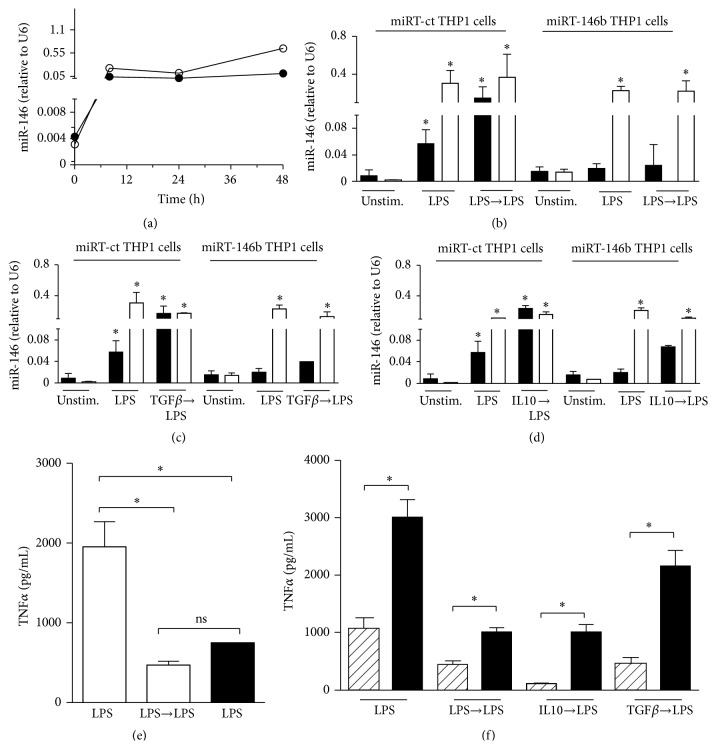
(a) THP-1 wild-type cells were stimulated for the indicated times with 1 *μ*g/mL of LPS. MiR-146b (black circles) and miR-146a (white circles) were measured by qPCR and their levels were normalized relative to U6. (b-d) miRT-ct and miRT-146b THP-1 cells were primed or not with 100 ng/mL LPS (b), 20 ng/mL IL-10 (c), or 50 ng/mL TGF*β* (d) for 18 h and then challenged with 1 ug/mL LPS for 24 h. MiR-146b (black columns) and miR-146a (white columns) were measured by qPCR and normalized to U6 levels. (e) TNF*α* protein levels were measured by ELISA in cell-free supernatants of pRRL-ct (white columns) or pRRL-146b (black columns) THP-1 cells primed or not with 100 ng/ml LPS and stimulated with 1 ug/mL LPS. (f) miRT-ct (white columns) and miRT-146b (black columns) THP-1 cells were primed or not with 100 ng/mL LPS, 20 ng/mL IL-10, or 50 ng/mL TGF*β* for 18 h and then challenged with 1 ug/mL LPS for 24 h. TNF*α* protein levels were quantified by ELISA. Results are expressed as mean ± SEM of three independent experiments.

**Figure 6:**
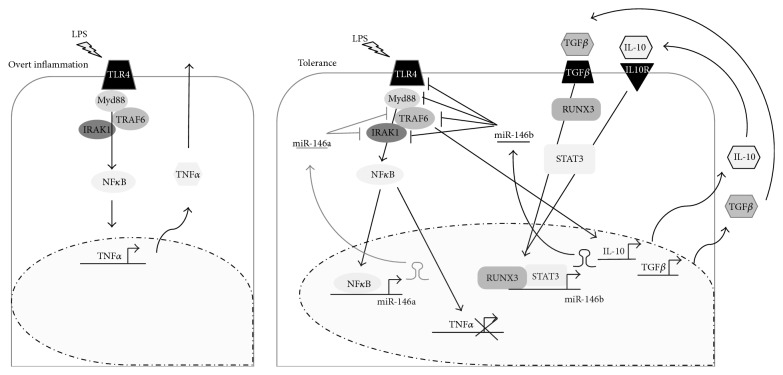
*MiR-146 a/b effect on induction of endotoxin tolerance.* During overt inflammation activation of the TLR4 signaling pathway triggers the induction of proinflammatory cytokines, including TNF*α*, IL-6, CCL3, and IL-12. In the late phase of the inflammatory response, the induction of anti-inflammatory genes, including IL-10 and TGF*β*, drives the expression of miR-146b through STAT3 and RUNX3, which negatively modulate TLR pathway at different steps, thus inducing tolerance.

To determine the role of miR-146b in endotoxin tolerance, we used the THP-1 monocytic cell line, a well-established model for* in vitro* study of endotoxin tolerance. We showed that both miR-146b and miR-146a were significantly induced by LPS (Figure 5(a)). This data were consistent with the expression data of miR-146b and miR-146a in human primary monocytes. In order to discriminate the relative contribution of miR-146b to the establishment of the endotoxin tolerance phenotype, we transduced THP-1 monocytic cells with lentiviral vectors to specifically overexpress (pRRL-146b THP-1 cells) or inhibit miR-146b isoform (miRT-146b THP-1 cells) without affecting miR-146a expression (Figures 5(b)–5(d)). As shown in Figure 5(e), pRRL-146b THP-1 cells stimulated with a single dose of LPS exhibited lower levels of TNF*α* as compared to pRRL-ct THP-1 cells, thus inducing a state of hyporesponsiveness to LPS similar to that observed by the subsequent LPS stimulation. Accordingly, the induction of endotoxin tolerance by priming monocytes with LPS, IL-10, or TGF*β* was completely abolished when the endogenous miR-146b expression was inhibited (Figure 5(f)). Therefore, LPS responsiveness was restored in all the three different* in vitro* model of endotoxin tolerance, demonstrating that miR-146b is induced during LPS tolerance as part of the inhibitory feedback mechanism that take place in monocytes to render cells refractory to subsequent LPS challenge.  Figure 6 depicts the proposal model of miR-146b and miR-146a effect on induction of endotoxin tolerance.

## 4. Discussion

Deregulation of the inflammatory response contributes to tissue damage in pathological conditions such as autoimmune diseases and cancer [3, 41, 42]. The innate immune system has developed a number of mechanisms, such as endotoxin tolerance, to balance inflammation. Endotoxin tolerance is a complex, orchestrated counter-regulatory response to inflammation, during which monocytes undergo a global transcriptional and functional reprogramming and enter into a refractory state, unresponsive to a subsequent LPS challenge. A characteristic feature of endotoxin tolerance is the downregulation of several proinflammatory genes and the concomitant upregulation of some anti-inflammatory genes [43]. In particular, the induction of IL-10 and TGF*β* in the late phase of the inflammatory response represents a key step for the induction of endotoxin tolerance [16, 21, 44] leading to leukocyte deactivation through the impairment of TLR signaling. Defects of TLR4 signaling have been observed at the level of the receptor, adaptors, transcription factors, and signaling molecules. In this regard, recent studies have focus attention on the differential expression and effects of miRNA in the modulation of TLR4 signaling in monocytes [18–20] and interestingly, new evidence suggests the involvement of miRNA in the regulation of key components of TLR4 pathway during endotoxin tolerance [28, 29].

The miR-146 family, composed by miR-146a and miR-146b, has been implicated in the regulation of immune cell signaling [45, 46] and more interestingly in the modulation of the inflammatory response in monocytes [18, 21, 22]. In particular, miR-146a was shown to be involved in LPS response and in endotoxin tolerance; recent clinical studies showed increased expression of miR-146a in monocytes isolated from CLL patients, challenged with LPS* ex vivo* [47]. A correlation between miR-146a gene polymorphisms and the risk of severe sepsis has also been described [48]. Despite these first studies highlighting the important role of miR-146a in endotoxin tolerance, the possible contribution of miR-146b in the regulation of the LPS response has not yet been addressed potentially based on the report by Taganov et al. [18], indicating that miR-146a was the major miRNA of the miR-146 family induced by LPS in THP-1 monocytes.

We previously described miR-146b as the other miR-146 family member able to negatively modulate the inflammatory response in phagocytes by targeting multiple components of the TLR signaling pathway [21]. Since defects in TLR signaling and in the consequent release of proinflammatory cytokines are characteristics of endotoxin tolerance, we thought to shed light on the potential role of miR-146b in the induction of the tolerant state. We first demonstrate that both miR-146b and miR-146a are expressed in LPS-stimulated human primary monocytes and in THP-1 monocytic cells. Secondly, their expression is induced in LPS-tolerized monocytes. Since both miRNAs are abundantly expressed in this cellular context and have overlapping target genes, to discriminate the relative contribution of miR-146b to the establishment of the endotoxin tolerance phenotype, we transduced THP-1 cells with a miR-146b lentiviral vector, which specifically inhibits miR-146b isoform without affecting miR-146a expression. We found that inhibition of endogenous miR-146b levels in LPS tolerized cells completely restored the levels of TNF*α* protein, here used as readout of endotoxin tolerance. Accordingly, the enforced expression of miR-146b in THP-1 monocytes mimicked the effects of LPS priming, inducing a state of tolerance comparable to that observed by the sequential stimulation with LPS in control cells. Remarkably, miR-146b but not miR-146a expression levels were further induced by LPS when monocytes were primed with TGF*β*- and IL-10-tolerized monocytes and this is consistent with the specific upregulation of miR-146b in anti-inflammatory conditions. Indeed, characterization of miR-146b transcriptional regulation by bioinformatics analysis and ChIP experiments showed that RUNX3, an important mediator of TGF*β* signaling, is specifically recruited to miR-146b promoter in monocytes challenged with TGF*β*, while STAT3 drives the expression of miR-146b in both TGF*β* and IL-10 stimulated monocytes. It is known that IFN*γ* can prevent or revert endotoxin tolerance through the inhibition of TGF*β* and IL-10 signaling [37], although the precise molecular mechanism responsible for IFN*γ*-dependent negative feedback loop is still incompletely elucidated. Importantly, the direct induction of miR-146b by IL-10 or TGF*β* as well as its indirect induction after LPS challenge was completely abolished by IFN*γ*.

Altogether our data propose miR-146b as a key anti-inflammatory miRNA, which acts together with miR-146a in mediating endotoxin tolerance. Notably, we demonstrated a differential regulation of miR-146b and miR-146a expression, suggesting that miR-146b could be one of the molecular tools used by IL-10 and TGF*β* to induce an LPS refractoriness state. We also suggest that the two miR-146 family members may represent the components of a relay team in which the two isoforms succeed each other to control expression of proinflammatory genes. Although the observed miR-146b effect in the induction of endotoxin tolerance is consistent with the previously shown regulation of the TLR signaling pathway by miR-146b [21], it is known that endotoxin tolerance is a more complex process, involving a wider cellular regulation. Other biological processes, such as phagocytosis or antigen presentation, should be investigated in* in vivo* models of endotoxin tolerance to shed light on the effective impact of miR-146b and miR-146a deregulation in the context of endotoxin tolerance. Moreover, due to the large clinical implications of endotoxin tolerance it would be interesting to test the impact of miR-146b deregulation in a clinical context. Further investigations covering all these aspects are needed to candidate miR-146b as a possible therapeutic tool for treating several clinical conditions, such as sepsis and other inflammatory-related disorders.
